# Doped Nanocrystalline Diamond Films as Reflective Layers for Fiber-Optic Sensors of Refractive Index of Liquids

**DOI:** 10.3390/ma12132124

**Published:** 2019-07-02

**Authors:** Monika Kosowska, Daria Majchrowicz, Kamatchi J. Sankaran, Mateusz Ficek, Ken Haenen, Małgorzata Szczerska

**Affiliations:** 1 Department of Metrology and Optoelectronics, Faculty of Electronics, Telecommunications and Informatics, Gdansk University of Technology, 11/12 Narutowicza Street, 80-233 Gdansk, Poland; 2 Institute for Materials Research (IMO), Hasselt University, Wetenschapspark 1, B-3590 Diepenbeek, Belgium; 3 IMOMEC, IMEC vzw, Wetenschapspark 1, B-3590 Diepenbeek, Belgium

**Keywords:** doped nanocrystalline diamond films, refractive index sensor, fiber-optic, nitrogen-doping, boron-doping, optical fiber sensor

## Abstract

This paper reports the application of doped nanocrystalline diamond (NCD) films—nitrogen-doped NCD and boron-doped NCD—as reflective surfaces in an interferometric sensor of refractive index dedicated to the measurements of liquids. The sensor is constructed as a Fabry–Pérot interferometer, working in the reflective mode. The diamond films were deposited on silicon substrates by a microwave plasma enhanced chemical vapor deposition system. The measurements of refractive indices of liquids were carried out in the range of 1.3 to 1.6. The results of initial investigations show that doped NCD films can be successfully used in fiber-optic sensors of refractive index providing linear work characteristics. Their application can prolong the lifespan of the measurement head and open the way to measure biomedical samples and aggressive chemicals.

## 1. Introduction

The field of biomedical measurements is rapidly growing, and optical sensors play a significant role in its development. The use of optical fibers in the construction of sensors provides many advantages [1]. Such sensors require only a small amount of sample, give a rapid response, measurements are non-invasive and chemical pretreatment is not needed [2]. Moreover, they assure no risk of electrical sparks and immunity to ionizing radiation [3]. This group of sensors is widely used for the determination of refractive index, one of the most important optical properties describing materials. The precise determination of its value allows for the identification of the investigated substance and its concentration [4,5]. Therefore, fiber-optic measurements of refractive index attracted considerable attention in finding applications in many fields including biomedicine, chemistry, environmental analysis and the food industry [6,7,8,9,10].

However, measurements of biological samples, hazardous and chemically aggressive substances are still a challenge. Investigation of such materials carries a high risk of damaging the elements of the measurement heads. In conventional Fabry–Pérot interferometers, the most exposed part is a mirror that has direct contact with the sample. In standard solutions it is made of metallic layers such as silver or aluminum. Even though they have satisfactory optical parameters, they are susceptible to mechanical damage and have limited chemical resistance. Aluminum can cause poisoning of the investigated tissues. This results in permanent damaging of the measurement head and the necessity of replacing the damaged part, which is inconvenient and causes additional costs. In performing measurements of biomedical substances, apart from the risk of damaging the sensor, there is a high chance of polluting the investigated sample, which can affect the results giving a false response. Thus, there is a need for new materials which can be used in the production of reflective surfaces that provide good optical parameters, as well as great resistance to chemicals. These features can be found in diamond materials.

Diamond films deposited in chemical vapor deposition (CVD) process are very hard, chemically inert and stable materials, which are transparent in a broad wavelength range [11,12,13]. The properties of diamond film structures produced in a CVD system can be tailored by changing the deposition process parameters. The use of dopants in the working gas mixture has an impact on the resulting film, including the optical properties. Hence it is possible to tune the process to achieve a suitable structure for the reflective layer in the interferometer [14,15,16].

In this work we present the application of diamond films doped with boron and nitrogen. The diamond films were deposited on silicon substrates and utilized as mirrors in fiber-optic sensors dedicated to measuring refractive indices of liquids. This solution combines the advantages of the fiber-optic Fabry–Pérot interferometer with benefits introduced by the extraordinary parameters of the diamond films, solving the problem of standard mirrors being susceptible to chemical damage.

This study is a continuation of our investigation regarding the use of diamond films in fiber-optic sensors. Previously, we proved that doped diamond films can be successfully applied in distance sensors and that they assure the biocompatibility [17,18]. This paper reports refractive index sensors utilizing two kinds of doped diamond films.

## 2. Materials and Methods

### 2.1. Nanocrystalline Diamond Films

Boron-doped nanocrystalline diamond (BD-NCD) films were synthesized in a microwave plasma enhanced CVD (MW PE CVD) system (SEKI Technotron AX5400S, Tokyo, Japan) on p-type Si (100) substrates. In our experiments, dedicated NCD suspensions were used to seed the substrates [19,20]. The pressure before growth in the vacuum chamber was kept at 10^−4^ Torr. A special truncated cone-shaped shim and 500 °C was used during the growth of BD-NCD films. The plasma microwave power, optimized for diamond synthesis, was kept at 1300 W [21]. In this study, the molar ratio of the CH_4_:H_2_ mixture was kept at 1% of gas volume at 300 sccm of the total flow rate. The boron level, expressed as the (B)/(C) ratio, in the gas phase was 10,000 ppm. The growth time was 3 h. After the growth process, the substrate temperature was slowly reduced (5 °C·min^−1^) down to room temperature.

Nitrogen-doped NCD (ND-NCD) film was grown on silicon substrate in a MW PE CVD system (SEKI ASTeX 6500, Tokyo, Japan). Prior to the diamond film growth, the silicon substrate was seeded with a colloidal suspension containing 5 nm detonation nanodiamonds and distilled water using a spin-coating technique. The ND-NCD film was grown on a silicon substrate in a CH_4_(9)/H_2_ (282)/N_2_(3) sccm plasma excited by 3000 W microwave power with 65 Torr pressure for 180 min. The substrate temperature was about 650 °C, which was measured using a single-color optical pyrometer.

The samples were made in two different universities. Two different pressures result from differences in the standard growth procedures developed in each scientific team. For convenient referencing to each sample, we adopted the following abbreviations: BD-NCD-Si—boron-doped nanocrystalline diamond deposited on a silicon substrate, and ND-NCD-Si—nitrogen-doped nanocrystalline diamond deposited on a silicon substrate.

#### Diamond Film Characterization

In order to be able to use a diamond film as a mirror, it has to fulfill specific requirements considering its surface. The film has to be homogenous, continuously covering the substrate. No cracks should be detected to provide reliable resistance to chemicals.

The diamond films used in this experiment were previously applied in fiber-optic sensors for determining their potential in measurements of biological samples and for distance measurements [14,22], hence, their detailed characterizations can be found there. The investigation has shown that the thickness of the doped NCD films is around 300 nm, and the root mean square roughness is equal to 22 nm and 49 nm for BD-NCD-Si and ND-NCD-Si, respectively [22]. The aforementioned examinations proved that the deposited diamond films can work as reflective surfaces.

### 2.2. Measurement Setup

**Figure 1:**
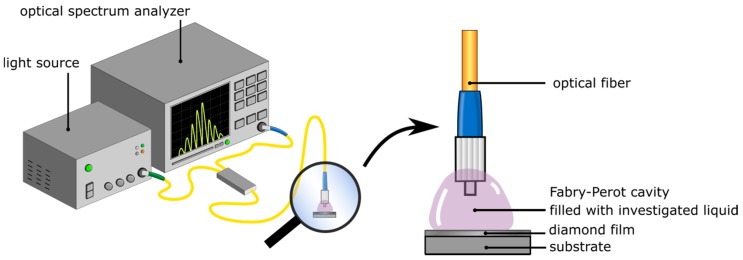
Measurement setup with a close-up of the measurement head.

The fiber-optic setup for measuring the refractive index of liquid samples based on a Fabry–Pérot interferometer was constructed. The experiment was carried out with two superluminescent diodes working at the central wavelength of 1290 nm and 1560 nm, respectively. The system was built with a light source (S-1290-G-I-20, and S1550-G-I-10, Superlum, Ireland), an optical spectrum analyzer (ANDO AQ6319, Yokogawa, Japan), single-mode telecommunication optical fibers (SMF-28, Thorlabs, Newton, NJ, USA), and a 2 × 1 50:50 coupler. The tip of the fiber and the doped NCD films were used as a measurement head. The principle of the operation of this setup was described elsewhere [22]. Figure 1 shows the schema of the measurement setup with a close-up of the measurement head.

**Figure 2:**
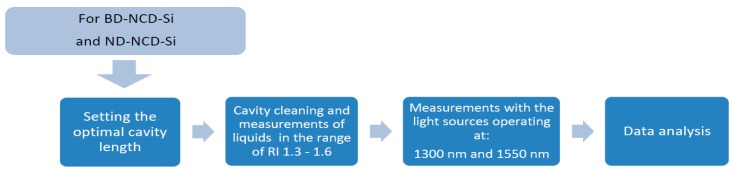
The block diagram of the experiment.

The measurement field, called a Fabry–Pérot cavity, is formed between the face of a fiber and the nanocrystalline diamond film deposited on silicon substrate. Measurements of refractive indices in the range of 1.3–1.6 were performed with the use of refractive index liquids provided by Cargille^®^. In the experiment and data analysis we considered the refractive indices of liquids at 1290 nm and 1560 nm according to the datasheets. However, in the text, we refer to each solution by a rounded value of its refractive index to provide greater clarity. Before placing the sample, the cavity was cleaned with isopropyl alcohol. The block diagram of the experiment is shown in Figure 2.

The distance between the fiber and the reflective surface in the device influences the visibility of the interferometric fringes, as the light beam diverges and is coupled back into the fiber with differing efficiency. Therefore, before carrying out refractive index measurements, the cavity length of the interferometer was set to achieve the highest visibility value of the measured signal. To calculate the visibility V, the following formula was used:

(1) $$V=\frac{I_{\max}-I_{\min}}{I_{\max}+I_{\min}}$$

where I_max_ is the maximum intensity of the measured signal and I_min_ is the minimum intensity of the measured signal. Table 1 shows values of visibility for the investigated films calculated for representative cavity lengths for given working wavelengths.

**Figure 3:**
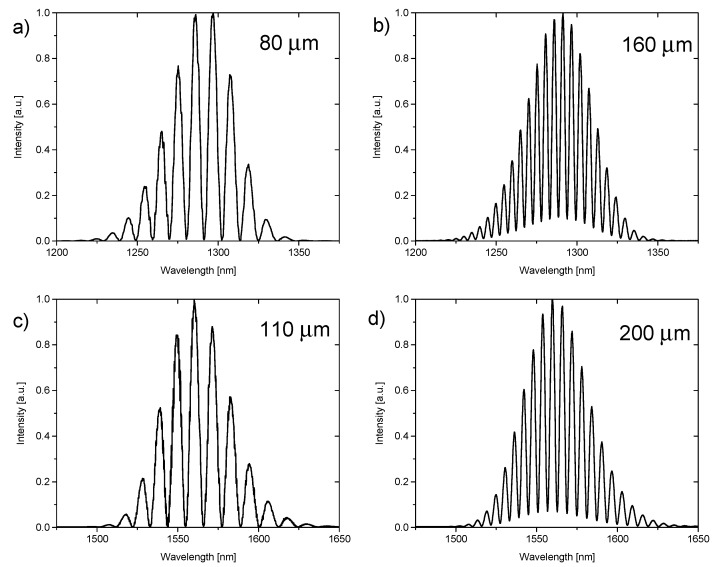
Spectra measured on BD-NCD-Si while the cavity was filled with air. Spectra obtained for 1290 nm and cavity lengths (**a**) 80 µm and (**b**) 160 µm. Spectra obtained for 1560 nm and cavity lengths (**c**) 110 µm and (**d**) 200 µm.

**Figure 4:**
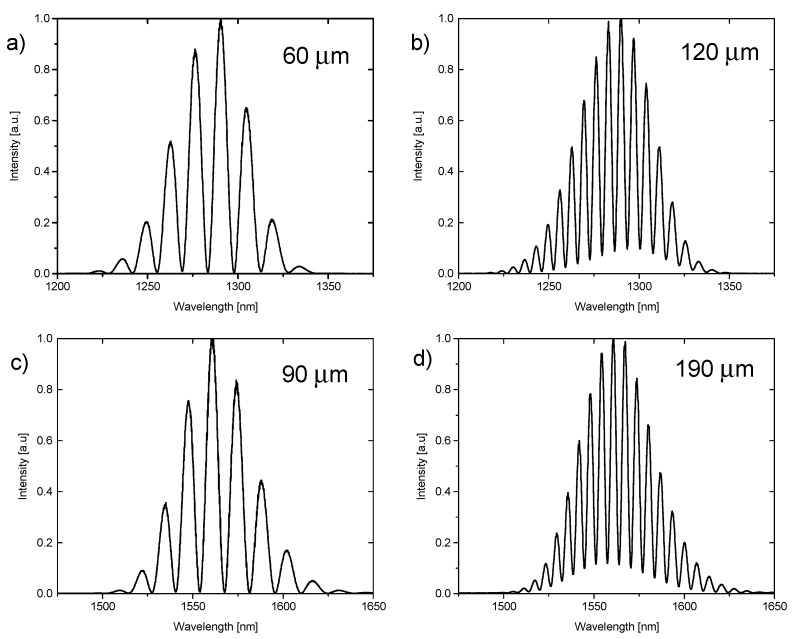
Spectra measured on ND-NCD-Si while the cavity was filled with air. Spectra obtained for 1290 nm and cavity lengths (**a**) 60 µm and (**b**) 120 µm. Spectra obtained for 1560 nm and cavity lengths (**c**) 90 µm and (**d**) 190 µm.

The above calculations of visibility were based on spectra presented in Figure 3 and Figure 4.

## 3. Results

**Figure 5:**
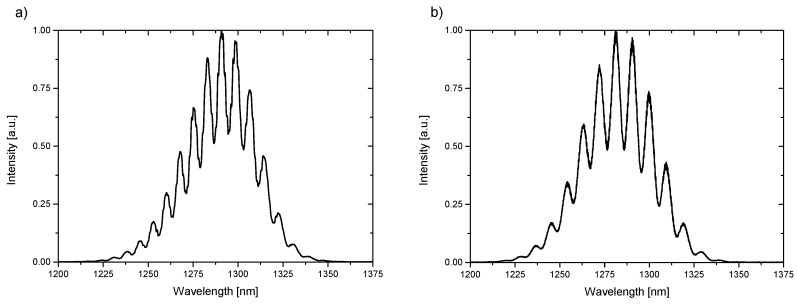
Spectra of liquid with refractive index n = 1.4 measured with a wavelength of 1290 nm (**a**) BD-NCD-Si, (**b**) ND-NCD-Si.

The measurement signals were recorded with the use of an optical spectrum analyzer. The representative spectra are shown in Figure 5.

**Figure 6:**
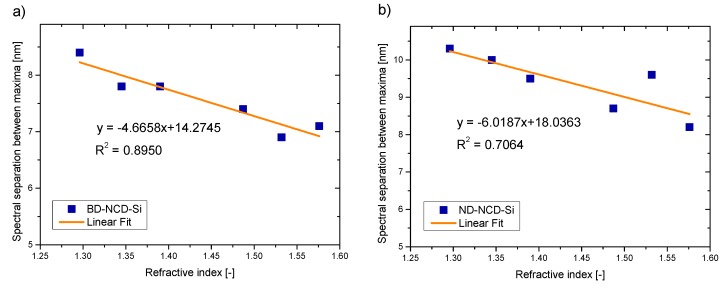
Measurement results: spectral separation between maxima as a function of refractive index for wavelength equal to 1290 nm (**a**) BD-NCD-Si, (**b**) ND-NCD-Si.

The obtained spectra were analyzed regarding the spectral separation between the maxima in the spectra for measured refractive indices of liquids. The results of these investigations for the light sources with a central wavelength equal to 1290 nm are presented in Figure 6.

The values of correlation coefficient R^2^ and sensitivity S for each sensor working at central wavelength of 1290 nm are presented in Table 2. The sensitivity can be directly found from the slope of the linear fit [9]. The values of R^2^ parameter in the range of 0.7–0.9 indicate high positive/negative correlation, while values higher than 0.9 mean very high positive/negative correlation [23].

**Figure 7:**
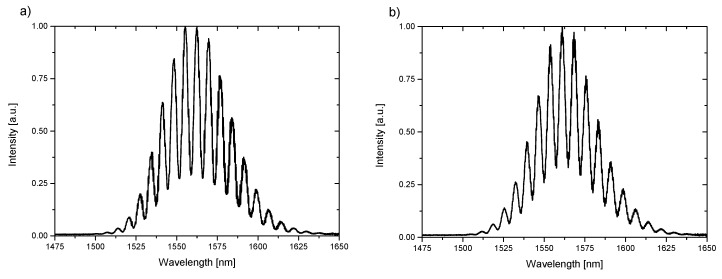
Spectra of liquid with refractive index n = 1.6 measured with a wavelength of 1560 nm (**a**) BD-NCD-Si, (**b**) ND-NCD-Si.

Similarly, the same procedure of data analysis was applied to the measurement data collected with the use of a source working at central wavelength of 1560 nm. The representative measurement spectra are shown in Figure 7.

**Figure 8:**
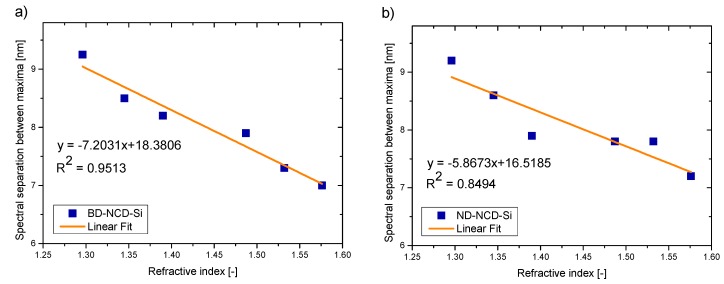
Measurement results: spectral separation between maxima as a function of refractive index for wavelength equal to 1560 nm (**a**) BD-NCD-Si, (**b**) ND-NCD-Si.

Figure 8 presents the relationship between the spectral separation of the interferometric fringes and the refractive index.

The values of correlation coefficient R^2^ and sensitivity S for each sensor working at central wavelength of 1560 nm are calculated and presented in Table 3.

It can be noted that all investigated samples can be applied as reflective surfaces in fiber-optic sensors of refractive index. The dependence between the spectral separation and refractive index is linear in each case, with a high or very high value of correlation coefficient R^2^.

The small reduction in sensitivity for the nitrogen-doped NCD can result from the lower value of reflectivity assured by this film in comparison with the boron-doped NCD film. The sensitivity is directly related to the reflection assured by the reflective film implemented in the sensor. The reflectivity of the surface is dependent on its refractive index. Reflectivity R at the boundary between the diamond film and the medium can be calculated by use of the Fresnel equation [24]:

(2) $$R={\left(\frac{n_{2}-n_{1}}{n_{1}+n_{2}}\right)}^{2}$$

where: n_1_, n_2_ are the refractive indices of the medium and NCD diamond film, respectively.

For the BD-NCD-Si sample, the value of the refractive index increases with the increase of wavelength, which results in a higher value of reflectivity. For the ND-NCD-Si sample, the value of the refractive index decreases with the increase of wavelength, which results in a lower value of reflectivity. The reflectivity for the BD-NCD-Si sample increased by 3.6% with the wavelength, while the reflectivity of the ND-NCD-Si sample decreased by 0.79%.

## 4. Conclusions

In this paper we reported the application of doped NCD films to fiber-optic interferometric sensors. The sensors are dedicated to the measurement of the refractive index of liquids. The experiments included NCD films doped with nitrogen and boron. The diamond films were produced using a microwave plasma enhanced chemical vapor deposition system. The investigation was performed in the refractive index range between 1.3–1.6. The linear mathematical models were adopted to the measurement data, allowing for determination of correlation coefficients R^2^ and sensitivity values for each sensor. The achieved values of R^2^ in the range of 0.7–0.9 and higher than 0.9 show high and very high negative correlation between the spectral separation of the fringes in the spectrum and the value of the refractive index. The initial study showed that the use of doped diamond films as reflective surfaces in sensors of refractive index is possible. This approach assures better resistance to chemical and mechanical damage prolonging the lifespan of sensors and allowing measurements of aggressive chemicals and biological samples.

## Figures and Tables

**Table 1:** Visibility values calculated for representative cavity lengths filled with air measured on BD-NCD-Si and ND-NCD-Si for central wavelength of 1290 nm and 1560 nm.

**BD-NCD-Si**			
**Wavelength—1290 nm**		**Wavelength—1560 nm**	
**Cavity Length**	**Visibility**	**Cavity Length**	**Visibility**
80 µm	0.9915	110 µm	0.9950
160 µm	0.8239	200 µm	0.8727
**ND-NCD-Si**			
**Wavelength—1290 nm**		**Wavelength—1560 nm**	
**Cavity Length**	**Visibility**	**Cavity Length**	**Visibility**
60 µm	0.9917	90 µm	0.9939
120 µm	0.8394	190 µm	0.7905

BD-NCD-Si: boron-doped nanocrystalline diamond deposited on a silicon substrate; ND-NCD-Si: nitrogen-doped nanocrystalline diamond deposited on a silicon substrate.

**Table 2:** Correlation coefficient (R^2^) and sensitivity (S) values for sensors working at 1290 nm.

Parameters	BD-NCD-Si	ND-NCD-Si
**R^2^**	0.8950	0.7064
**S (nm/a.u.)**	−4.6658	−6.0187

**Table 3:** Correlation coefficient (R^2^) and sensitivity (S) values for sensors working at 1560 nm.

Parameters	BD-NCD-Si	ND-NCD-Si
**R^2^**	0.9513	0.8494
**S (nm/a.u.)**	−7.2031	−5.8673
